# Case Report: Rare occurrence of *Pseudomonas aeruginosa* osteomyelitis of the right clavicle in a patient with IgA nephropathy

**DOI:** 10.12688/f1000research.3891.1

**Published:** 2014-11-06

**Authors:** Aishwarya Damodaran, Anusha Rohit, Georgi Abraham, Sanjeev Nair, Anand Yuvaraj

**Affiliations:** 1 Madras Medical Mission Hospital, Chennai, 600037, India

## Abstract

We describe the case of a 47 year old patient with proven primary IgA nephropathy who presented with osteomyelitis of the medial end of the right clavicle. The patient was not on immunosuppressive medications. He underwent aspiration curettage and CT scan of the clavicle which yielded pus that grew *Pseudomonas aeruginosa*. Following treatment with appropriate antibiotic therapy the patient presented a complete recovery of the lesion with no loss of renal function. This case highlights the importance of positive cultures in the choice of the appropriate therapy in an extremely rare case of an immunocompetent patient with osteomyelitis of the clavicle.

## Introduction

Osteomyelitis of the clavicle is an extremely rare occurrence with an incidence of less than 1% in mixed age population, with *Staphylococcus aureus* being the most commonly isolated organism ^1, 2^. Patients often have a history of immunosuppression or invasive procedures such as tracheostomy or subclavian vein catheterisation. Here we report the case of a 47 year old man with IgA nephropathy who developed osteomyelitis of the medial end of right clavicle caused by *Pseudomonas aeruginosa*.

## Case description

**Figure 1.**
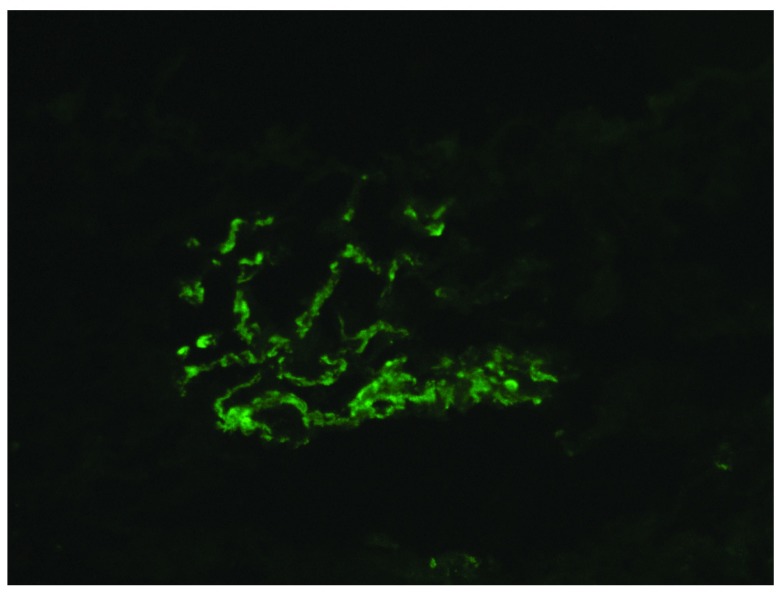
Immunofluorescence showing strong positive for IgA in the capillary loop and mesangium.

**Figure 2.**
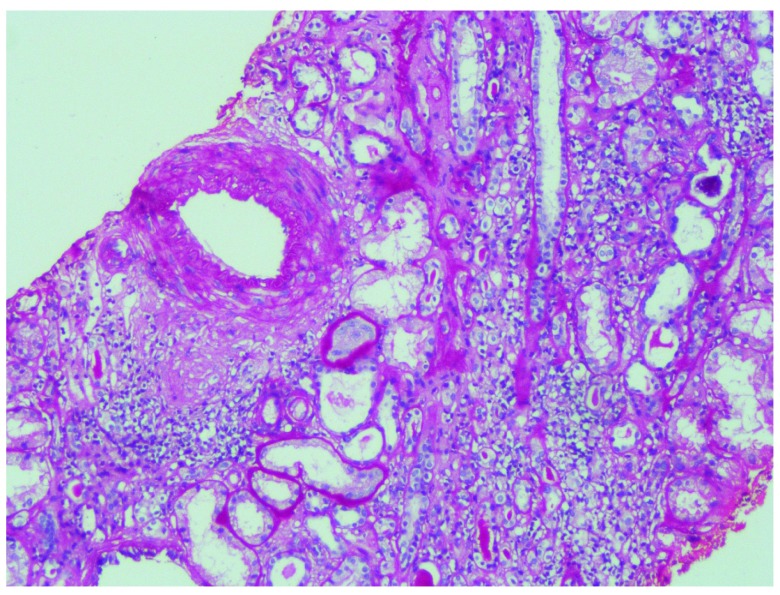
Hematoxylin and eosin staining showing tubular atrophy, interstitial fibrosis and thickened blood vessel.

A 47 year old South Asian male teacher presented to our institute in June 2012 with a diagnosis made elsewhere of accelerated hypertension and acute left ventricular failure following a recent anterior wall myocardial infarction. He was admitted to hospital for further cardiac management, and a nephrology consultation was sought for renal insufficiency (serum creatinine 1.9 mg/dl). The presence of an active urinary sediment (urine albumin 3+ by dipstick and microscopic hematuria) necessitated a renal biopsy which showed IgA nephropathy ( Figure 1) with an Oxford Pathological score of M1E0S1T1, 5/10 glomerular sclerosis and 30% IF/TA with hypertensive changes in blood vessels ( Figure 2). The patient was initiated on olmesartan 20 mg/day and prednisolone 50 mg/day and a follow up for management of proteinuria was advised.

He presented again 7 months later with swelling pain and erythema of the right clavicle that had been present for 2 months. He was evaluated for these complaints at another centre before presentation to ours and was diagnosed with synovitis of the medial end of the right clavicle. Ultrasound guided aspiration of fluid from the swelling and analysis of bacterial cultures in an external laboratory did not reveal any organism. The patient was subsequently treated with non steroidal anti inflammatory drugs and prednisolone for 1 week.

Physical examination following admission at our centre revealed a normal body mass index (BMI) and vitals with an otherwise unremarkable systemic examination. Local examination of the right clavicle revealed a swelling of 2×3 cm with erythema, induration, warmth and tenderness with a firm consistency. The skin did not show discharging sinuses. Laboratory investigations revealed the following: white blood cells (WBC) count 12,400 cells/mm ^3^ with predominant polymorphonuclear cells, platelet count 290,000/mm ^3^, erythrocyte sedimentation rate (ESR) 93 mm/hr, urea 42 mg/dl and serum creatinine 1.5 mg/dl. Liver function tests revealed: serum glutamic oxaloacetic transaminase (SGOT) 69 IU, serum glutamic-pyruvic transaminase (SGPT) 44 IU, alanine aminotransferase (ALP) 130 kU, total protein 7.3 g/dl serum albumin 3.3 g/dl. Serology for HIV, hepatitis B and the venereal disease laboratory test (VDRL) were negative, anti-streptococcal antibody (ASO) was titre-negative, and C3 and C4 levels were within the normal range.

Urine analysis showed 1+ albumin by dipstick, and microscopic examination of the urine showed 6–8 WBC/high power field (hpf), 2–3 epithelial cells/hpf, 4–6 RBCs/hpf and occasional granular casts. Sonography of the abdomen showed bilateral normal sized kidneys with increased echogenicity and maintained corticomedullary differentiation. Ultrasound guided aspirate of the pus from the right clavicle was cultured using BacT Alert 120 (bioMerieux, France) which showed Gram-negative and oxidase-positive rods. This suggested the presence of *P. aeruginosa* that was found to be sensitive to cefoperazone and sulbactam, using Vitek Compact II (bioMerieux, France). Repeated staining for Acid Fast Bacilli was negative. Tuberculosis (TB) PCR analysis and staining for fungal elements were negative. CT scan of the clavicle showed cortical and subcortical irregularity with soft tissue swelling of the medial end of right clavicle suggestive of osteomyelitis. Curettage and lavage of the site was followed by a gentamicin impregnated dressing (Septocoll E, Biomet Deutschland GmbH, Berlin), and histopathological examination of the sequestrum revealed non-caseating granulomas surrounded by many neutrophils, lymphocytes and plasma cells, suggestive of chronic osteomyelitis. The patient was begun on intravenous cefoperazone and sulbactum 1.5 g BD as per the sensitivity report for a period over 3 weeks.

## Discussion

Osteomyelitis most commonly involves the metaphyses of long bones and its occurrence in the clavicle as a primary infection is extremely rare ^1^. The incidence of clavicular osteomyelitis in mixed adult population is less than 1% with 7% incidence in paediatric population ^3^, and in adults is almost always due to prior trauma or invasive procedures in close proximity to the sternoclavicular area ^4^. Examples of medical procedures that can cause osteomyelitis include tracheostomy, sternotomy or subclavian vein catheterisation, and osteomyelitis is often associated with immunosuppression therapy. Even in such cases the most common organism causing infection in more than 95% of the cases is *S. aureus*. In this case *P. aeruginosa* was the organism identified ^5^.

*P. aeruginosa* is an aerobic, motile, non-fermenting Gram-negative bacillus which produces a biofilm. It is usually regarded as a trivial commensal of the skin, mucosa and intestinal tract, but on occasions it can be the cause of severe hospital-acquired infections. Osteoarticular infections generally include pelvic and vertebral infections in patients with primary or secondary immunodeficiency, prior to prolonged broad spectrum antibiotic therapy, vascular insufficiency, intravenous drug abuse or other invasive procedures ^6, 7^ and, when secondary to Pseudomonas spp, are associated with a greater risk of recurrence and amputation ^6^. Our patient had an Ig A nephropathy treated with low dose steroid therapy for only a short while (1 week). He presented evidence of chronic renal changes such as tubular atrophy, interstitial fibrosis and blood vessel changes as shown by the biopsy. As there was no other secondary cause detected we presumed that this was a primary IgA nephropathy. The natural course of IgA nephropathy ranges from benign non-progressive disease to end stage renal failure which occurs in 15–40% of the patients over a span of 10–20 years ^7^.

The presence of osteomyelitis did not lead to rapid deterioration of renal function, as appropriate diagnosis and antimicrobial treatments were initiated without significant delay. As our patient had insignificant proteinuria, it is possible that his renal function deterioration would be slow as compared to patients with IgA nephropathy and heavy proteinuria.

There have been reports of reversals of secondary IgA nephropathies which developed post-chronic osteomyelitis after treating the infection ^8^. But in this case, the diagnosis of IgA nephropathy was made 7 months prior to the onset of osteomyelitis. In this patient, there was no evidence to suggest a correlation between IgA nephropathy and profound renal insufficiency causing immunosuppression which led to osteomyelitis.

In conclusion, we report a rare occurrence of osteomyelitis of the clavicle due to *P. aeruginosa* in a non-immunosuppressed patient with chronic kidney disease and primary IgA nephropathy.

## Consent

Informed written consent for publication of clinical details was obtained from the patient.

## References

[1] Calhoun JH Manring MM Shirtliff M: Osteomyelitis of the long bones. *Semin Plast Surg.* 2009;23(2):59–72. 10.1055/s-0029-1214158 20567728 PMC2884908

[2] Carlos GN Kesler AK Coleman JJ: Aggressive surgical management of sternoclavicular joint infections. *J Thorac Cardiovasc Surg.* 1997;113(2):242–247. 10.1016/S0022-5223(97)70319-2 9040616

[3] Piazza C Magnoni L Nicolai P: Clavicular osteomyelitis: a rare complication after surgery for head and neck cancer. *Eur Arch Otorhinolaryngol.* 2006;263(7):653–6. 10.1007/s00405-006-0040-z 16612610

[4] Judich A Haik J Rosin D: Osteomyelitis of the clavicle after subclavian vein catheterization. *JPEN J Parenter Enteral Nutr.* 1998;22(4):245–246. 10.1177/0148607198022004245 9661128

[5] Balakrishnan C Vashi C Jackson O: Post-traumatic osteomyelitis of the clavicle: A case report and review of literature. *Can J Plast Surg.* 2008;16(2):89–91. 19554172 10.1177/229255030801600208 PMC2691560

[6] Tice AD Hoaglund PA Shoultz DA: Risk factors and treatment outcomes in osteomyelitis. *J Antimicrob Chemother.* 2003;51(5):1261–1268. 10.1093/jac/dkg186 12668581

[7] Muñoz-Fernández S Maciá MA Pantoja L: Osteoarticular infection in intravenous drug abusers: influence of HIV infection and differences with non drug abusers. *Ann Rheum Dis.* 1993;52(8):570–574. 10.1136/ard.52.8.570 8215617 PMC1005112

[8] Donadio JV Grande JP: IgA nephropathy. *N Eng J Med.* 2002;347(10):738–748. 10.1056/NEJMra020109 12213946

[9] Tevlin MT Wall BM Cooke CR: Reversible renal failure due to IgA nephropathy associated with osteomyelitis. *Am J Kidney Dis.* 1992;20(2):185–8. 10.1016/S0272-6386(12)80549-X 1496974

