# Supplementary material for: Case Report: Rare occurrence of Pseudomonas aeruginosa osteomyelitis of the right clavicle in a patient with IgA nephropathy
Source: F1000Res. 2014 Nov 6;3:268. [Version 1] doi: 10.12688/f1000research.3891.1 (PMC4275023; doi:10.12688/f1000research.3891.1)
Supplement: Peer review history

# Peer review: Referee response for version 1

**DOI:** 10.5256/f1000research.4167.r7007

**Published:** 2014-12-11

**Authors:** Kashi Nath Prasad

**Affiliations:** 1 Department of Microbiology, Sanjay Gandhi Post Graduate Institute of Medical Sciences, Uttar Pradesh, India

**General comments**

Osteomyelitis of right clavicle in an imuunocompetent individual is a rare entity that too by an opportunistic bacterium like *Pseudomonas aeruginosa*. Here the authors report osteomyelitis of medial end of clavicle caused by P. aeruginosa in an immunocompetent individual with IgA nephropathy. The case is interesting and it highlights the importance of prompt diagnosis of such cases and initiation of appropriate antibiotics.

**Specific comments**

It is not clear if the patient had any history of trauma or any invasive procedure on clavicle? Ultra sound guided aspiration was done in another hospital that did not yield any bacterial growth. Could infection by *P. aeruginosa* be iatrogenic because of earlier aspiration procedure?

*P. aeruginosa *was found sensitive to cefoperazone and sulbactum but what about the sensitivity to other antibiotics. Authors should provide the antibiotic profile of the isolate.

Could it be possible that IgA nephropathy and marked renal failure led to immunosuppressed state precipitating osteomyelitis?

I have read this submission. I believe that I have an appropriate level of expertise to confirm that it is of an acceptable scientific standard.

# Peer review: Referee response for version 1

**DOI:** 10.5256/f1000research.4167.r6662

**Published:** 2014-11-12

**Authors:** K V Dakshinamurthy

**Affiliations:** 1 Department of Nephrology, Nizam's Institute of Medical Sciences, Hyderabad, India

This is an interesting and informative article. It however needs clarification regarding the statement that this patient is non-immunosuppressed. This patient is a patient of chronic kidney disease, which by itself is an immunosuppressed state. He received prednisolone at a dose of 50 mg per day after the diagnosis of IgA nephropathy. The duration of therapy was not mentioned. Prednisolone therapy produces a state of immunosuppression. He presented again 7 months later with swelling pain and erythema of the right clavicle that had been present for 2 months. Therefore the infection occurred in an immunosuppressed individual.

I have read this submission. I believe that I have an appropriate level of expertise to confirm that it is of an acceptable scientific standard.
